# The Role of Gastrointestinal Microbiota in Functional Dyspepsia: A Review

**DOI:** 10.3389/fphys.2022.910568

**Published:** 2022-06-08

**Authors:** Li Zhou, Yi Zeng, Hongxing Zhang, Yan Ma

**Affiliations:** ^1^ Department of Rehabilitation Medicine, Wuhan Hospital of Integrated Traditional Chinese and Western Medicine, Wuhan, China; ^2^ Department of Hospital Infection Management Office, Wuhan Hospital of Integrated Traditional Chinese and Western Medicine, Wuhan, China; ^3^ Department of Acupuncture, Wuhan Hospital of Integrated Traditional Chinese and Western Medicine, Wuhan, China

**Keywords:** functional dyspepsia, gastrointestinal microbiota, dysbiosis of the gastrointestinal microbiota, intestinal mucosal barrier, mucosal immunity

## Abstract

Functional dyspepsia is a clinically common functional gastrointestinal disorder with a high prevalence, high impact and high consumption of medical resources. The microbiota in the gastrointestinal tract is a large number of families and is one of the most complex microbial reservoirs in the human body. An increasing number of studies have confirmed the close association between dysbiosis of the gastrointestinal microbiota and the occurrence and progression of functional dyspepsia. Therefore, we reviewed the role of dysbiosis of the gastrointestinal microbiota, *H. pylori* infection and gastrointestinal microbiota metabolites in functional dyspepsia, focusing on the possible mechanisms by which dysbiosis of the gastrointestinal microbiota contributes to the pathogenesis of functional dyspepsia. Several studies have confirmed that dysbiosis of the gastrointestinal microbiota may cause the occurrence and progression of functional dyspepsia by disrupting the biological barrier of the intestinal mucosa, by disturbing the immune function of the intestinal mucosa, or by causing dysregulation of the microbial-gut-brain axis. Probiotics and antibiotics have also been chosen to treat functional dyspepsia in clinical studies and have shown some improvement in the clinical symptoms. However, more studies are needed to explore and confirm the relationship between dysbiosis of the gastrointestinal microbiota and the occurrence and progression of functional dyspepsia, and more clinical studies are needed to confirm the therapeutic efficacy of microbiota modulation for functional dyspepsia.

## Introduction

Functional dyspepsia (FD) is one of the most common types of functional gastrointestinal diseases (FGIDs) in clinical practice, with a high prevalence affecting 10%–30% of adults and 3.5%–27% of children worldwide (Drago et al., 2021). The main clinical symptoms of patients are early satiety, postprandial discomfort, epigastric pain, epigastric distension, epigastric burning, loss of appetite, belching, nausea, and vomiting, which are often accompanied by anxiety and depression (Potter and Talley, 2020; Zand et al., 2021). It not only affects the life and work of patients, but also brings economic pressure to patients and national medical services. According to the Rome IV diagnostic criteria, FD refers to the presence of the above symptoms, but no gastrointestinal organic or structural lesions explained by gastroenteroscopy, ultrasound, computed tomography, gastrointestinal barium meal examination and other examinations (Wei et al., 2020). Clinically, FD can be divided into epigastric pain syndrome (EPS), postprandial distress syndrome (PDS), and EPS-PDS overlap group. Among them, patients with EPS have epigastric pain and epigastric burning as the main clinical symptoms, and patients with PDS have postprandial fullness and early satiety as the main clinical symptoms. In our Asian countries, patients with PDS type are the most common (Asano et al., 2017).

According to existing studies, the occurrence of FD is associated with gastrointestinal motility disorders, increased visceral sensitivity, impaired gastric tolerance, impaired gastrointestinal mucosal integrity, abnormal function of brain-gut axis, increased eosinophils in duodenum, dysbiosis of the gastrointestinal microbiota, *Helicobacter-pylori* (*H. pylori*) infection, post-gastrointestinal infection, diet, genetics, mental and psychological factors (Madisch et al., 2018; Black et al., 2020; Ford et al., 2020). Currently, gastrointestinal motility agents, anti-anxiety and depression drugs, *H. pylori* eradication drugs, Chinese acupuncture and Chinese herbal medicine are also used to treat FD in clinical practice (Kang et al., 2019; Ford et al., 2021; Ho et al., 2021; Kwon et al., 2021). The human digestive tract is the largest reservoir of microbiota in the body, so it is easily affected by the microbial ecological environment. In recent years, an increasing number of studies have confirmed that dysbiosis of the gastrointestinal microbiota plays an important role in the occurrence and progression of FD (Tziatzios et al., 2020; Lee, 2021).

## Gastrointestinal Microbiota

The gastrointestinal microbiota is an important component of the human body. The normal human gastrointestinal tract contains more than 1,000 species and more than 100 trillion microbes. These microbiota reside in the human gastrointestinal tract and play an important role in maintaining the gastrointestinal barrier, immune and metabolic functions (Barko et al., 2018). The human gastrointestinal tract has the most dense and complex microbiota pool in the body, and these gastrointestinal microbiota are interdependent with the human body, forming a mutually beneficial relationship; and they contain more than 100 times the total number of human genes, making it a large gene pool worthy of study. The huge number of the gastrointestinal microbiota in the gastrointestinal tract in a certain proportion to achieve a dynamic balance of species and number. Proteobacteria, Firmicutes, Actinobacteria and Bacteroidetes were the main microorganisms isolated from human gastrointestinal tract, accounting for more than 98% of the total number of the gastrointestinal microbiota (Huang et al., 2019). The fecal and intestinal biopsy tissues of healthy subjects showed a predominance of the Firmicutes, followed by the Actinobacteria and Bacteroidetes (Vaga et al., 2020). Once this balance is disrupted, a series of pathological changes can occur due to dysbiosis of the gastrointestinal microbiota. In recent years, the correlation between the gastrointestinal microbiota and human health and diseases has become one of the hot spots, and more and more studies have shown that the occurrence and progression of many human diseases are more or less related to the dysbiosis of the gastrointestinal microbiota, such as inflammatory bowel disease (Lloyd-Price et al., 2019), FD (Tziatzios et al., 2020), irritable bowel syndrome (Shin et al., 2019) and other gastrointestinal diseases, hypertension (Hsu et al., 2019) and other cardiovascular diseases (Brown and Hazen, 2018), respiratory diseases such as asthma (Huang and Boushey, 2015), Alzheimer’s disease (Borsom et al., 2020), malignant tumors (Saus et al., 2019), diabetes (Gou et al., 2021), etc.

## Gastrointestinal Microbiota and Its Metabolites and FD

### Gastrointestinal Microbiota and FD

Proteobacteria phylum belongs to Gram-negative bacteria, which is the largest phylum of bacteria and one of the most abundant phyla in the human gastrointestinal microbiota. Its name comes from Proteus, the god capable of shape-shifting in ancient Greek mythology. Proteobacteria phylum is divided into five classes, *α*, *β*, *γ*, *δ,* and *ε*, according to rRNA sequences, and the bacteria are highly heterogeneous and can present different morphologies. Within humans, Proteobacteria phylum exists not only in the gastrointestinal tract, but also in the skin, mouth, vagina and other parts, which can be symbiotic bacteria or pathogenic bacteria. An increased abundance of Proteobacteria is a sign of dysbiosis of the gastrointestinal microbiota and can be used as a potential diagnostic criterion for diseases (Shin et al., 2015). Most of the bacteria in Firmicutes phylum belongs to the Gram-positive bacteria, and it’s named for the thicker cell wall of most of the bacteria in the phylum, which is often spherical or rod-shaped under the microscope. Firmicutes phylum can be divided into three classes: anaerobic *Clostridium*, facultative or aerobic *Bacillus*, and non-cell wall Hymenomycetes. Actinobacteria phylum is also Gram-positive bacteria, named for the radiating growth of their colonies, which has a higher GC content than Firmicutes phylum. Bacteroidetes phylum is abundant in the gastrointestinal tract of humans and animals, accounting for more than 60% of the total gastrointestinal microbiota of the digestive tract. And it can be divided into three classes, Bacteroidetes, Flavobacteria and Sphingobacillaceae. Bacteroidetes phylum is involved in the fermentation of carbohydrate, the utilization of nitrogenous substances and the biotransformation of bile acids and other steroids in the human gastrointestinal tract.

Proteobacteria, Firmicutes, Actinobacteria, and Bacteroidetes are known as the “four phyla” of the gastrointestinal microbiota in the human body (Rizzatti et al., 2017). Once their relative abundance or composition proportion in the gastrointestinal tract is abnormal, it will cause dysbiosis of the gastrointestinal microbiota and lead to diseases. In recent years, the development of research technologies such as analytical biology, 16SrDNA high-throughput sequencing, and gene sequencing has facilitated the study of the gastrointestinal microbiota and increased the heat on the correlation between the gastrointestinal microbiota and human health and disease. Table 1 summarizes data from studies investigating gastrointestinal microbiome alterations in FD.

**TABLE 1:** Gastrointestinal microbiome analysis studies in functional dyspepsia.

Ref	Species	Number (FD/controls, n)	Technique for microbiota identification	Principal findings
Qiu et al. (2017)	Rats	3/3	16S rDNA V4 gene sequencing	Lower abundance of the bacteroidetes phylum, higher abundance of the proteobacteria and firmicutes phylum
Zhang et al. (2020)	Mice	12/12	16S rRNA gene sequencing	Down regulation of bacteroidetes, *Lactobacillus*, and prevotellaceae, up regulation of proteobacteria, verrucomicrobia, epsilonbacteraeota, firmicutes, lachnospiraceae NK4A136 group, and lachnospiraceae
Zhong et al. (2017)	humans	9/9	16S rRNA gene sequencing	Lower abundance of actinomycete, atopobium collin, leptotrichia trevisan, prevotella, and veillonella, The total relative abundance of bacteria was positively correlated with the severity of clinical symptoms
Fukui et al. (2020)	humans	11/7	16S rRNA V3-V4 gene sequencing	Up regulation of the phylum Firmicutes and *Streptococcus*, The relative abundance of *Streptococcus* was positively correlated with upper gastrointestinal symptoms
Nakae et al. (2016)	humans	44/44	16S rDNA gene sequencing	Lower abundance of Prevotella, higher abundance of Bifidobacterium and *Clostridium*, The relative abundance of Prevotella was negatively correlated with the severity of PDS symptoms

In one study, sequencing of feces from rats in a liver-depression and spleen-deficiency model of FD using 16S rDNA high-throughput sequencing technology revealed that the relative abundance of the Bacteroidetes phylum in the feces of rats with FD was significantly decreased compared to healthy rats, whereas the relative abundance of the Proteobacteria and Firmicutes phylum was significantly increased (Qiu et al., 2017). In another experimental animal study, it was also found that the relative abundance of Bacteroidetes, *Lactobacillus* and Prevotellaceae in the gastrointestinal tract of FD mice decreased, while the relative abundances of Proteobacteria, Verrucomicrobia, Epsilonbacteraeota, Firmicutes, Lachnospiraceae NK4A136 group, and Lachnospiraceae increased (Zhang et al., 2020). In addition, 16SrRNA sequencing of duodenal mucosal flora of 9 FD patients and nine healthy subjects showed that *Streptococcus* was the most abundant in the duodenal mucosa of both FD patients and healthy subjects, but there was a significant decrease in the relative abundance of Actinomycete, Atopobium Collin, Leptotrichia Trevisan, Prevotella, and Veillonella in the duodenal mucosa of FD patients compared to normal subjects. Moreover, the total relative abundance of bacteria was positively correlated with the severity of clinical symptoms of the patients (Zhong et al., 2017). In one study, FD patients were found to have significantly increased the phylum Firmicutes and *Streptococcus* in the upper gastrointestinal tract compared to healthy subjects, and the relative abundance of *Streptococcus* was positively correlated with upper gastrointestinal symptoms (Fukui et al., 2020). In another study, it was shown that FD patients had reduced abundance of Prevotella and increased abundance of Bifidobacterium and *Clostridium* compared to healthy subjects, and that the relative abundance of Prevotella was negatively correlated with the severity of PDS symptoms (Nakae et al., 2016).

In conclusion, it is evident that the disturbance in the relative abundance and composition of the microbiota in the gastrointestinal tract is important for the process of FD occurrence and progression. In addition, different segments of gastrointestinal tract contain different microbiota. Studies have shown that human colon segments are dominated by anaerobic bacteria such as Bacteroidetes and Lachnospiraceae, while small intestine segments are dominated by parthenogenic anaerobic bacteria (Frank et al., 2007). Compared with healthy controls, FD patients not only had different gastrointestinal microbiota, but also had different oral microbiota abundance and composition. Proteobacteria were the dominant bacteria in FD patients’ saliva, while Bacteroidetes were the dominant bacteria in healthy controls. According to 16SrRNA sequencing of saliva, the abundance of Spirochaetes in FD patients was higher than that in healthy controls, while the abundance of Fusobacteria, TM7 and Proteobacteria was lower than that in healthy controls, and the levels of Kingella and Abiotrophia genus levels were also significantly different (Liu et al., 2021).

### Gastrointestinal Microbiota Metabolites and FD

The gastrointestinal microbiota has a complex metabolic process in the human body. It not only provides itself with the necessary energy for growth and reproduction, but also can use the intestinal contents and the endogenous mucus secreted by the intestinal epithelium to produce a variety of metabolites, including short-chain fatty acids (ScFAs), cholic acid, choline metabolites, phenols, lipids, carbohydrates, etc. These metabolites may be harmful or beneficial to the human body, and are closely related to human health and the occurrence and progression of many diseases. Table 2 summarizes data from studies investigating metabolites of the gastrointestinal microbiota.

**TABLE 2:** Studies of gastrointestinal microbiota metabolites and their effects.

Ref	Metabolites	Primary sources	Effects
Markowiak-Kopeć and Śliżewska, (2020); Serpa et al., (2010); Haveaar, (2011)	ScFAs (formic acid, acetic acid, propionic acid, butyric acid)	The *Clostridium* group of Firmicutes phylum, and *Lactobacillus*, Bifidobacterium, Eubacteriaceae, Fecal bacteria	Regulation of the pH value in the intestine; Promoting the absorption of water, sodium, calcium, magnesium and other substances; Inhibiting the multiplication and growth of pathogenic bacteria and the activity of intestinal inflammatory mediator; Maintaining the integrity of gap junctions in the intestine.
Alexandrov et al. (2020)	Lipid (cholesterol, LPS, peptidoglycan)	Bifidobacterium, *Lactobacillus*, Enterobacteriaceae and *Clostridium*	Regulation of the intestinal permeability and intestinal immunity; Disruption of the body’s immune system and induction of inflammatory responses.
Brown et al. (2019)	Lipid (sphingolipids)	Bacteroidetes and Prevotellaceae	Aggravating intestinal inflammation
Gao et al., (2019); Gao et al. (2020)	Indole-derived (tryptophan)	*Clostridium* sporogenes and *Escherichia coli*	Regulation of the brain-gut axis and protection against stress-induced damage in the gastrointestinal tract

The metabolites of ScFAs include formic acid, acetic acid, propionic acid, butyric acid, etc. Formic acid is less abundant in the intestinal tract, while the content of the latter three accounts for more than 90% of the total ScFAs in the intestinal tract (Ríos-Covián et al., 2016). ScFAs are mainly produced by the *Clostridium* group of Firmicutes phylum, as well as by *Lactobacillus*, Bifidobacterium, Eubacteriaceae and Fecal bacteria using some dietary fiber, resistant starch, oligosaccharides and other compounds that are not easy to digest in the intestinal tract (Markowiak-Kopeć and Śliżewska, 2020). Acetic acid is the metabolite produced by fermentation of most bacteria, propionic acid is the main metabolite of Bacteroidetes, and butyric acid is the main metabolite of Firmicutes. Studies have shown that ScFAs can regulate the pH value in the intestine, promote the absorption of water, sodium, calcium, magnesium and other substances, and provide more than 70% of the energy for the intestinal epithelial cells, especially butyric acid (Serpa et al., 2010). ScFAs are also able to inhibit the multiplication and growth of pathogenic bacteria and the activity of intestinal inflammatory mediators, thus playing an anti-inflammatory role in the intestinal tract (Havenaar, 2011). In recent years, studies on the pathogenesis of FD have shown that the damage of duodenal mucosal barrier and immune activation played an important role in the pathogenesis of FD, and more than 40% of FD patients had microinflammatory cell infiltration in the duodenum (Nojkov et al., 2020). In addition, gastrointestinal motility disorders is an important pathogenesis of FD. And interstitial cells of Cajal (ICCs) are the pacemaker cells of gastrointestinal tract, which can form SIP syncytium structures with surrounding platelet-derived growth factor receptor α-positive cells (PDGFRα^+^) and smooth muscle cells (SMCs) through tight intercellular gap junctions (Baker et al., 2013). It can also form a nerve fibers-ICCs-SMCs network with the surrounding enteric nervous system and SMCs through tight intercellular gap junctions, which plays an important role in regulating the pacing and slow wave propagation of the gastrointestinal tract (Sanders et al., 2014). Studies have shown that ScFAs are able to maintain the integrity of gap junctions in the intestine.

Lipid metabolites include cholesterol, lipopolysaccharide (LPS), peptidoglycan, and sphingolipids, which are mainly produced by Bifidobacterium, *Lactobacillus*, Enterobacteriaceae and *Clostridium*. Studies have shown that lipid metabolites can affect intestinal permeability and intestinal immunity. LPS is generally released by the death lysis of Gram-negative bacteria, and it stimulates tumor necrosis factor alpha (TNFα), interleukin-1β (IL-1β), interferon-gamma (IFNγ), interleukin-8 (IL-8) and other inflammatory factors are released to disrupt the body’s immune system and induce inflammatory responses (Alexandrov et al., 2020). Sphingolipids can be produced by the intestinal symbiotic bacteria Bacteroidetes and Prevotellaceae. It has been found in animal studies that sphingolipids can also aggravate intestinal inflammation (Brown et al., 2019). Indole-derived metabolites are produced by the fermentation of *Clostridium* sporogenes and *Escherichia coli*. Such metabolites are able to participate in the regulation of gastrointestinal disorders by regulating the brain-gut axis and protecting against stress-induced damage in the gastrointestinal tract. Tryptophan is a key monoamine neurotransmitter involved in the regulation of central neurotransmission and intestinal physiological functions, and studies have shown that the gastrointestinal microbiota can regulate the brai006E-gut axis through tryptophan metabolism (Gao et al., 2019; Gao et al., 2020).

## *H. Pylori*, Gastrointestinal Microbiota and FD

*H. pylori* is a Gram-negative that colonizes mainly the stomach and duodenum and interacts with the gastrointestinal microbiota. *H. pylori* colonization in the stomach and duodenum can cause changes in pH and mucosal damage in the stomach and duodenum, which can further cause colonization by other bacteria, leading to changes in the gastrointestinal microbiota. Sequencing of 16S rRNA genes showed significant differences in the abundance of bacteria on the gastric and duodenal mucosa between *H. pylori*-positive and *H. pylori*-negative subjects, with *H. pylori*- patients having significantly higher levels of *Helicobacter*, while the relative abundance of the phylum Actinobacteria, Bacteroidetes, Firmicutes and *Clostridium* were significantly lower (Schulz et al., 2018). Another study showed that *H. pylori* infection can cause changes in the relative abundance of *Neisseria*, Rothia, TM7-3, Leptotrichia, Lachnospiraceae, Megasphaera, F16, Moryella, Filifactor, and Paludibacter bacteria and disrupt the normal colonization of the duodenum (Maeda et al., 2022). *H. pylori* infection can also mediate the body’s immune response, thus affecting the gastrointestinal microbiota. It was found that *H. pylori*-positive patients had significantly different abundance of microbiota in the stomach compared to *H. pylori*-negative subjects, and their serum Foxp3, interleukin-10 (IL-10), and transforming growth factor-β (TGF-β) levels were increased, consistent with increased T-regulatory cell responses (Brawner et al., 2017). In addition, the microbiota in the gastrointestinal tract can also affect the colonization of *H. pylori*, and studies have found that *Lactobacillus* can inhibit the growth of *H. pylori* strains and can be used in the treatment of *H. pylori* infections (Salas-Jara et al., 2016; Huang et al., 2022). Studies have shown a significant correlation between *H. pylori* infection and the composition of the gastrointestinal microbiota, particularly Prevotella copri and Eubacterium biforme (Lapidot et al., 2021).

Previous studies have confirmed that there is a causal relationship between *H. pylori* infection and dyspeptic symptoms, and that *H. pylori* infection is an important pathological factor in the occurrence and progression of FD, and its specific mechanism of action may be related to inflammation of the gastrointestinal mucosa and altered gastrointestinal motility (Kim et al., 2017; Koletzko et al., 2019). A follow-up of *H. pylori*-positive people revealed that patients with a history of *H. pylori* infection were at higher risk of developing FD (Loor et al., 2021). In contrast, *H. pylori* eradication treatment is effective in improving symptoms in patients with *H. pylori*-associated dyspepsia (Tanaka et al., 2021). *H. pylori* infection has the ability to affect not only the microbiota in the gastrointestinal tract but also the microbial metabolism, which affects the occurrence and progression of FD through these pathways (White et al., 2021).

## Possible Mechanisms of FD Due to the Dysbiosis of Gastrointestinal Microbiota

### Disruption of the Intestinal Mucosal Barrier

The normal intestine is protected by a biological barrier that separates the intestinal luminal contents from the internal environment of the organism and prevents the invasion of foreign bacteria, which is called the intestinal mucosal barrier. It is a semi-permeable barrier that allows the absorption and transport of nutrients but not the entry of harmful substances, luminal antigens and pathogens, and plays an important role in a variety of physiological functions such as digestion, absorption and metabolism (Farré et al., 2020). The composition of the intestinal mucosal barrier includes the mucus layer of the intestine, intestinal epithelial cells, microbiota in the intestine and antimicrobial peptides secreted by intestinal epithelial cells, etc. The mucus layer is located in the innermost layer of the intestinal cavity, which on the one hand can facilitate the downward movement of the intestinal contents down the intestine, and on the other hand separates the intestinal contents from the intestinal epithelial cells, which can protect the intestinal epithelium from acids, digestive enzymes, and pathogenic bacteria in the intestinal lumen (Paone and Cani, 2020). When the abundance and composition of the gastrointestinal microbiota are altered, the number of beneficial bacteria in the intestine decreases and the number of pathogenic bacteria increases, and pathogenic bacteria, as well as the endotoxins released by them, can invade the intestinal mucosa, which can damage the intestinal mucosal barrier and cause increased permeability of the intestine. Studies have shown that the gastrointestinal microbiota can affect the barrier function of the intestinal mucosa directly by stimulating the proliferation of epithelial cells or inducing the secretion of cytokines by epithelial cells, and indirectly by synthesizing essential nutrients, vitamins and ScFAs, which are energy sources for intestinal epithelial cells.

Damage to the intestinal mucosal barrier can cause increased permeability of the intestinal mucosa and reduced blockage of harmful substances by the intestinal mucosal barrier, which is one of the important mechanisms in the pathogenesis of FD (Taki et al., 2019). In a pilot study using measurement of baseline impedance to assess the integrity of the small intestinal mucosa, baseline impedance was found to be significantly lower in the duodenum and jejunum of patients with dyspepsia compared to healthy controls, indicating that patients with dyspepsia have impaired small intestinal mucosal integrity and increased permeability (Nakagawa et al., 2020). In addition, tight junction proteins play an important role in the barrier function of the duodenal mucosa, and tight junction proteins such as ZO-1 and CX43 are commonly used as indicators to assess the barrier function and permeability function of the intestinal epithelial mucosa (Oshima and Miwa, 2016). Studies show that monobutyric acid and monovaleric acid in ScFAs can upregulate the expression of the tight junction protein ZO-1 and thus protect the intestinal mucosal barrier (Nguyen et al., 2020). Intestinal epithelial cells are an important component of the intestinal mucosal barrier and are differentiated from stem cells at the base of the intestinal mucosal crypts, and the maintenance and repair of the intestinal mucosal barrier depends on the normal proliferation and differentiation of these stem cells (You et al., 2021). The Wnt/β-catenin signaling pathway is a key regulator of intestinal epithelial stem cells and plays an important regulatory role in the proliferation and maintenance of intestinal epithelial stem cells (Koch, 2017). Studies have shown that Citro Bacter and *Salmonella* can cause excessive proliferation of stem cells at the base of the intestinal mucosal crypts in mice and instead disrupt the intestinal epithelial barrier, whereas *Lactobacillus* reuteri and *Lactobacillus* acidophilus can protect the integrity of the intestinal epithelial barrier and maintain the intestinal mucosal barrier function by modestly regulating the proliferation of intestinal epithelial cells and increasing the secretion of antimicrobial peptides through activation of the Wnt/β-catenin signaling pathway (Wu et al., 2020).

### Disturbance of the Intestinal Immunity

Another important role of the gastrointestinal microbiota is to shape the intestinal immune response as well as to initiate systemic innate immunity. Recent studies have confirmed that immune activation of the duodenal mucosa plays an important role in the development and progression of FD, with mild inflammatory cell infiltration of the duodenum present in upwards of 40% of FD patients (Nojkov et al., 2020). Dysbiosis of the gastrointestinal microbiota can cause abnormalities in the intestinal barrier and function, which can activate the intestinal immune response and trigger gastrointestinal diseases such as FD. A one-year follow-up of patients with gastroenteritis caused by *Salmonella* infection revealed a significant increase in the probability of FD (Mearin et al., 2005), and an increase in the number of eosinophils and mast cells was evident in the gastric mucosa and duodenal mucosa of FD patients after infection (Kindt et al., 2009). An animal study showed that gut flora feeding germ-free mice drove the development of B lymphocytes and T lymphocytes in mice, and also reduced interleukin-4 (IL-4) production and increased interleukin-12 (IL-12), IL-10 and IFNγ production (Hrncir et al., 2008). Another study showed that nuclear factor-κB (NF-κB) is a key regulator of immune function and has an important role in the pathogenesis of autoimmune diseases as well as inflammatory diseases (Sun, 2017). Activation of the Akt/NF-κB signaling pathway can lead to the release of inflammatory factors in the intestinal barrier, resulting in intestinal inflammation. In contrast, the intestinal microbial metabolites ScFAs can inhibit NF-κB transfer and suppress the secretion of inflammation-related factors interleukin-2 (IL-2), interleukin-6 (IL-6), and TNF-α.

In addition, small intestinal bacterial overgrowth (SIBO) characterized by an increased concentration of bacteria in the small intestine, is also an important mechanism in the pathogenesis of FD (Gurusamy et al., 2021). Usually, the bacterial concentration in the small intestine is lower than 103 colony forming units (CFU)/mL, and the bacterial species is predominantly Gram-positive. Bacterial concentrations higher than 105 CFU/ml in the small intestine are diagnosed as SIBO, and also in the intestine of patients with the presence of SIBO, bacterial species are found to be altered, often with large numbers of Gram-negative and anaerobic bacteria (Losurdo et al., 2020). Studies have shown that there are two possible mechanisms by which SIBO triggers FD: one may be the direct damage to the integrity of the intestinal mucosa by overgrown bacteria, which can lead to increased intestinal mucosal permeability, the other may be the activation of the intestinal immune response by metabolites produced by these bacteria, causing the release of inflammatory factors or immune mediators that trigger the intestinal inflammatory response (Gasbarrini et al., 2007; Lauritano et al., 2010).

### Dysregulation of the Microbial-Gut-Brain Axis

The physiological activity of the gastrointestinal tract is regulated by the enteric nervous system, the central nervous system and the autonomic nervous system in multiple ways, among which there is a bidirectional regulation mechanism between the gut and the brain. The interaction between the microbiota in the intestine, the intestine and the central nervous system constitutes the microbial-gut-brain axis, which refers to the phenomenon that changes in the microbiota in the intestine can cause changes in various physiopathological activities in the intestine, and then transmit the stimuli to the central nervous system, which in turn can regulate various physiopathological activities in the intestine. Under stress conditions, alterations in central nervous system activity can also regulate gastrointestinal motility, immunity, secretion, and other functions as well as affect the composition of the gastrointestinal microbiota (Wang and Kasper, 2014). ScFAs, metabolites of the gastrointestinal microbiota, are often considered as key mediators of communication between the central nervous system and the intestine, and ScFAs induce intestinal secretion of glucagon-like peptide 1 (GLP1), γ- aminobutyric acid (GABA), and other hormones that can transmit stimuli to the central nervous system via the circulatory system or the vagal pathway (Silva et al., 2020). In addition, the gastrointestinal microbiota such as *Bacteroides*, Bifidobacterium, *Escherichia coli* can also overproduce neurotransmitters such as GABA (Strandwitz et al., 2019).

In one study, it was found that feeding with a high-fat diet can lead to dysbiosis of the gastrointestinal microbiota and stimulate Toll-like receptors 4 (TLR4) inflammation on microglia by increasing LPS ectopic and activating LPS-binding proteins (LBP) activation of the pathway (Jamar et al., 2021). In another animal study, TLRs expression was elevated and gastrointestinal motility was decreased after stimulation of mice using antimicrobial drugs, suggesting that the gastrointestinal microbiota can regulate gastrointestinal motility function stimulating neuroimmunity through activation of TLRs inflammatory pathway (Grasa et al., 2015).

### Other Mechanisms

The process of FD is closely related to factors such as gastrointestinal motility disorders and gastrointestinal hypersensitivity, which in turn are closely related to the gastrointestinal microbiota. 5-Hydroxytryptamine (5-HT), as an immunomodulatory factor, is present in large quantities in the gastrointestinal tract and is involved in the regulation of gastrointestinal motility and sensation (Fu et al., 2019). Dysbiosis of the gastrointestinal microbiota and some of the metabolites it produces can lead to an increase in the number of mast cells in the intestinal mucosa, thus causing the release of active substances such as 5-HT and histamine in the gastrointestinal tract, which are involved in the regulation of gastrointestinal dynamics and visceral sensitivity. It has been shown that the increased abundance of Prevotella, *Lactobacillus* and Alistipes in the intestine is positively correlated with the concentration of saturated long-chain fatty acids (SLCFAs), and the increased concentration of SLCFAs in the intestine can promote the contraction of intestinal smooth muscle, which causes increased intestinal motility (Zhao et al., 2018). The injection of bacteria into germ-free rats induces an increase in gastrointestinal slow-wave activity.

In addition, there was a correlation between the gastrointestinal microbiota and the visceral pain pathway, where the presence of inflammatory stimuli in germ-free mice was found to diminish pain perception, while the use of probiotics effectively improved the pain response in mice; indicating that the presence of the gastrointestinal microbiota can influence the onset and progression of gastrointestinal disease processes not only by affecting gastrointestinal myoelectric activity, but also by affecting visceral nociception, among other pathways (Chichlowski and Rudolph, 2015).

## Potential Treatment for FD: Modulating Microbiota

Since dysbiosis of the gastrointestinal microbiota is closely related to the occurrence and progression of FD, regulation of the gastrointestinal microbiota becomes one of the potential therapeutic modalities for FD. In a randomized controlled trial using probiotics (*Bacillus coagulans* MY01 and *Bacillus subtilis* MY02) versus placebo for the treatment of patients with FD, the efficacy of the treatment group with probiotics was significantly higher than that of the placebo group, but there was no significant difference between the efficacy of patients in the placebo group who were also using proton pump inhibitors and the treatment group (Wauters et al., 2021). In another clinical randomized controlled trial, treatment of FD with a mixture of probiotics (*Bacillus coagulans*, *Bacillus clausii*, and *Bacillus subtilis*) was significantly more effective than the placebo group in improving a variety of clinical symptoms such as eructation, bloating, belching, and acid reflux in patients (Soman and Swamy, 2019). It was also found that dietary treatment with a protein formula supplemented with *Lactobacillus rhamnosus* was effective in preventing FGIDs in children with milk allergy (Nocerino et al., 2019). The Extra-virgin olive oil diet, which is rich in probiotics, is also effective in improving the digestive symptoms of FD patients (Ianiro et al., 2013). A large number of studies have confirmed that probiotics can effectively regulate the gastrointestinal microbiota and are safe and effective for the treatment of FD, which may provide a potential mechanism for the clinical treatment of FD. In addition, probiotics have been shown to inhibit *H. pylori* and thus may improve *H. pylori*-associated dyspepsia symptoms, but some studies have also found that probiotics are still effective in improving symptoms in FD patients with *H. pylori*-uninfected (Ohtsu et al., 2017).

## Conclusion

**FIGURE 1:**
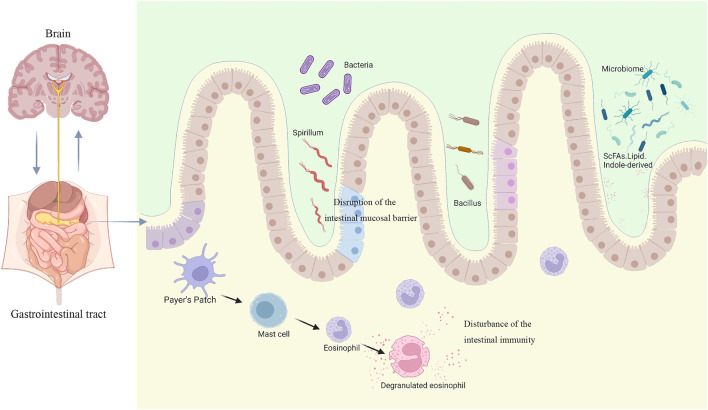
Disease models for the pathogenesis of FD associated with gastrointestinal microbiota (created with BioRender.com).

The mechanism of FD due to dysbiosis of the gastrointestinal microbiota mainly includes two situations: on the one hand, the abnormal composition and abundance of the gastrointestinal microbiota itself causes gastrointestinal tract dysfunction, and on the other hand, the change of metabolites due to the alteration of the gastrointestinal microbiota leads to abnormal gastrointestinal tract function. A large number of basic and clinical studies have shown that there are dysbiosis of the gastrointestinal microbiota such as decreased diversity and abundance of the gastrointestinal microbiota in FD patients and animals, especially the decrease of relative abundance of *Firmicutes phylum*, which dominates gastrointestinal microbiota and plays an important regulatory role in maintaining immune homeostasis in the intestine. In addition, abnormal metabolites of the gastrointestinal microbiota can also disrupt the intestinal biological barrier and immune barrier. Both dysbiosis and abnormal metabolites of the gastrointestinal microbiota can mediate the occurrence and progression of gastrointestinal diseases by disrupting the intestinal mucosal barrier, disturbing the intestinal immune function, and causing dysregulation of the microbial-intestinal-brain axis (Figure 1).

Since dysbiosis of the gastrointestinal microbiota is one of the pathological mechanisms in the pathogenesis of FD, probiotics and *H. pylori* eradication drugs are commonly used in clinical treatment to treat FD. A large number of studies have confirmed that *H. pylori* eradication drugs can effectively inhibit the activity of *H. pylori* in the gastrointestinal tract, while probiotics can effectively regulate the microbiota in the gastrointestinal tract and inhibit the growth of harmful bacteria, thus protecting the barrier function of the gastrointestinal mucosa and immune function and effectively preventing and treating the occurrence of FGIDs (Padole et al., 2021; Wauters et al., 2021). Currently, probiotics have been widely used in the clinical treatment of FD, irritable bowel syndrome (Moeen-Ul-Haq et al., 2022), functional constipation (Kim et al., 2021), functional diarrhea (Jung et al., 2022) and other gastrointestinal diseases, and probiotics have been effective in the clinical treatment of these FGIDs.

The gastrointestinal microbiota in human body is influenced by many factors, including daily diet, living habits, drug consumption, genetics, environmental factors, stress factors, etc. These factors have a certain influence on the structure and composition of the gastrointestinal microbiota. And the gastrointestinal microbiota in humans varies with age, and studies have shown that the intestinal tract of newborns is dominated by the colonization of parthenogenic anaerobic bacteria such as Enterobacteriaceae, *Streptococcus* and *Lactobacillus*, while after 1 week of life the intestinal tract is dominated by anaerobic bacteria such as Bifidobacterium, *Clostridium* and *Bacteroides*, but when they begin to add complementary foods, the gastrointestinal microbiota also begins to become diverse and relatively stable (Grier et al., 2017). Due to the individual variability of the gastrointestinal microbiota, both techniques and tools are currently challenging for humans to study the interactions between host diseases and the gastrointestinal microbiota.
